# Unique clinical and electrophysiological features in the peripheral nerve system in patients with sialidosis – a case series study

**DOI:** 10.1186/s13023-024-03216-8

**Published:** 2024-05-24

**Authors:** Sung-Ju Hsueh, Chin-Hsien Lin, Ni-Chung Lee, Tung-Ming Chang, Sung-Pin Fan, Wan-De Huang, Yea-Huey Lin, Li-Kai Tsai, Yin-Hsiu Chien, Ming-Jen Lee, Wuh-Liang Hwu, Hsueh Wen Hsueh, Chih-Chao Yang

**Affiliations:** 1 Department of Neurology, Yunlin County, National Taiwan University Hospital Yunlin Branch, 579 Sec. 2 Yunlin Road, Douliu, Taiwan; 2 Department of Neurology, National Taiwan University Hospital, No. 7 Chung-Shan South Road, Taipei, 10002 Taiwan; 3 Department of Medical Genetics, National Taiwan University Hospital, 7 Chung-Shan South Road, Taipei, Taipei, Taiwan; 4 Department of Pediatrics, Changhua Christian Hospital, 320 Hsu-Kuang Road, Changhua, Taiwan; 5 Department of Neurology, National Taiwan University Hospital Hsin-Chu Branch, Hsinchu County, 2, Sec. 1, Shengyi Road, Zhubei City, Taiwan; 6 Department of Anatomy and Cell Biology, National Taiwan University College of Medicine, 1 Renai. Road, Taipei, Taiwan

**Keywords:** Sialidosis, Hyperexcitability, Peripheral nerve, Very late response

## Abstract

**Background:**

To investigate the peripheral nervous system involvement in _*S*_ sialidosis with typical features of myoclonus, seizure, and giant waves in somatosensory evoked potentials suggesting hyperexcitability in the central nervous system.

**Methods:**

The clinical presentation of patients with genetically confirmed sialidosis was recorded. Neurophysiological studies, including nerve conduction studies (NCSs), F-wave studies, and needle electromyography (EMG), were performed on these patients.

**Results:**

Six patients (M/F: 2:4) were recruited. In addition to the classical presentation, intermittent painful paresthesia was noted in four patients, and three of whom reported it as the earliest symptom. In the NCSs, one patient had reduced compound muscle action potential amplitudes in the right ulnar nerve, while another patient had prolonged distal motor latency in the bilateral tibial and peroneal nerves. Prolonged F-wave latency (83.3%), repeater F-waves (50%), and neurogenic polyphasic waves in EMG (in 2 out of 3 examined patients) were also noted. Interestingly, a very late response was noted in the F-wave study of all patients, probably indicating lesions involving the proximal peripheral nerve or spinal cord.

**Conclusion:**

In addition to the central nervous system, the peripheral nervous system is also involved in sialidosis, with corresponding clinical symptoms. Further study on these phenomena is indicated.

**Supplementary Information:**

The online version contains supplementary material available at 10.1186/s13023-024-03216-8.

## Introduction

Sialidosis is an autosomal recessive disease with pathogenic variants in the NEU1 gene [1]. It is a rare disease with an estimated prevalence of 1 in 4 million births [2]. Sialidosis encompasses different symptoms, including myoclonus, seizures, ataxia, and visual disturbance, with variated onset ages [3, 4]. Myoclonus is induced by various minor stimulations, such as sound stimuli, light touch, and passive and voluntary movements. Cherry-red spots in the macula are often [4] – but not universally [5–7] –observed in patients, along with degeneration of the retina.

The pathogenesis has been proposed to be reduced neuraminidase activity leading to sialyloligosaccharide accumulation in affected tissues [1]. The reduced neuraminidase activities result in oversialylated lysosomal membrane protein 1, which in turn results in excessive exocytosis of lysosome contents. Pathologically, neurons in the central nervous system may show excessive sialyloligosaccharide accumulation in the cytoplasm. Radiologically, diffuse cerebral atrophy may be observed, with the cerebellar region prone to involvement, although this finding is not universal.

Involvement of the central nervous system (CNS) in sialidosis, presented as hyperexcitability and disinhibition, had been well studied [8, 9]. However, the involvement of the peripheral nervous system (PNS) has rarely been reported. Here we report the clinical and electrophysiological features in the PNS among patients with sialidosis.

## Results

We recruited six nonconsanguineous patients with genetically-confirmed sialidosis from unrelated Taiwanese families; two of the patients are siblings (Case 5 &6). Four patients were female. The mean age at initial disease presentation was 10.3 ± 5.2 years. The clinical phenotypes, genotype, enzyme activity assays, and electrophysiological findings are summarized in Tables 1 and 2, and Table 3. Among the typical symptoms of sialidosis, five patients had myoclonus at the time of evaluation, while ataxia or cerebellar signs were noted in four patients. Hyperreflexia and spasticity were observed in all patients. Four patients had cherry red spots. Among the four patients who received brain MRI, only one patient presented with mildly delayed myelination near both the frontoparietal central parenchyma and both posterior internal capsules. Four patients (Case 1 since the age of 21 years old, Case 2 since the age of 18, Case 3 since the age of 24, Case 6 since the age of 21) were wheelchair-bound, and the other two patients still walk independently on the examinations. (Case 4: 18 years old, Case 5: 33 years old).

**Table 1:** Clinical profiles of patients with sialidosis

Case/sex/age of onset (y)	Initial Presentation	Additional clinical presentation	Cherry Red spot	Genetic/Enzyme activity	Current management
1/F/5	Painful paresthesia, reluctance to move	Myoclonus, seizure, Hyperreflexia, ataxia	Present	c.544 A > G (p.Ser182Gly), c.640 C > T 0.012 nmol/mg prot/hr	Clonazepam, levetriacetam, piracetam, perampanel, acetazolamide
2/F/3	Painful paresthesia	Ataxia, myoclonus, seizure, hyperreflexia	Present	c.590 C > G (p.A197G), c.1153del"AGGCCCCCCAGCTCTACGTCCT”	Piracetam, clonazepam, perampanel
3/F/11	Painful paresthesia	Myoclonus, seizure, cerebellar sign	Present	c.544 A > G (p.Ser182Gly), c.667_679del(p.Leu223GlufsTer76) 0.09 nmol/mg Prot/hr	Clonazepam, levetriacetam, valproic acid
4/M/10	Painful paresthesia	Myoclonus, seizure	Present	c.544 A > G (p.Ser182Gly), c.314_352del (p.A106_G118del) 0.0323 nmol/mg Prot/hr	Propranolol, clonazepam, levetriacetam
5/F/18	Unsteady gait	Myoclonus, ataxia, Hyperreflexia	Not present	c.544 A > G (p.Ser182Gly), homozygote 0.11 nmol/mg Prot/hr	Levetriacetam
6/M/around 15	Unsteady gait	Ataxia, dysarthria	Not present	c.544 A > G (p.Ser12182Gly), homozygote	Levetriacetam

**Table 2:** Electrophysiological studies in patients with sialidosis

Case/sex/age of onset (y)	Age of exam (y)	EEG	Evoked potentials	Nerve conduction studies	Late response		Electromyogram
					F-wave, A-wave, H-reflex	Presence of very late response	
1/F/5	14	-Frequent myoclonic seizures with time-locked muscle contraction -An episode of generalized tonic-clonic seizure recorded	-Prolonged VEP latency -Giant wave in SSEPs	Normal	Normal	Yes	Normal
2/F/3	14	-Sharpened slow waves with phase reversal at C3 -Transient bursts of polyspike-and-wave discharges in the bilateral frontocentral areas	-Prolonged VEP and SSEP latency -Giant wave in SSEPs	Normal	-Decreased F-wave persistence in the bilateral median nerves, absence of F-waves in the bilateral tibial and peroneal nerves -Prolonged H reflex latency in right tibial nerve	Yes	Diffuse neurogenic waves without denervation changes
3/F/11	22	-Frequent generalized polyspike-and-wave complexes, -Frequent myoclonic seizures with time-locked muscle contraction	-Absence of VEP -Giant wave in SSEP, prolonged lower limb latency	Reduced CMAP amplitude in right ulnar nerve	-Prolonged F wave latency in bilateral peroneal and tibial nerves -A-waves in right tibial nerves -Repeater F morphology in right peroneal and left median nerves	Yes	Not performed
4/M/10	17	Normal	-Prolonged VEP -Giant wave in SSEP, prolonged lower limb latency	Normal	-Prolonged F-wave latency in bilateral tibial and peroneal nerves	Yes	Not performed
5/F/18	25	Normal	-Giant wave in SSEPs, prolonged lower limb latency	Prolonged distal motor latency in bilateral tibial and peroneal nerves	-Prolonged F-wave latency in bilateral tibial and peroneal nerves -Repeater F waves present	Yes	Diffuse neurogenic waves without denervation changes
6/M/around 15	28	Normal	-Prolonged VEP -Giant wave in SSEPs, prolonged upper and lower limb latency	Normal	-Prolonged F-wave latency in bilateral median, tibial and peroneal nerves	Yes	Not performed

**Table 3:** Findings from nerve conduction studies in patients with sialidosis*

Case		CMAP amplitude (mV)	Motor NCV (cm/s)	SAP amplitude (µV)	Sensory NCV (cm/s)	F-wave minimal latency (ms)	F-wave persistence	Very late response
Case 1	Median	11.2	56	103	64	25.9	66.7%	
	Ulnar	10.4	63	53	64	25.9	100%	
	Tibial	17.6	43			46.8	100%	Present
	Peroneal	3.6	44			46.4		
	Sural			21	48		66.7%	
Case 2	Median	10.9	44	99	61	30.0	100%	
	Ulnar	8.2	48	84	58	27.6	10%	
	Tibial	14.0	40			Absence		Present
	Peroneal	6.1	41			Absence		
	Sural			55	48			
Case 3	Median	10.7	59	66	58	28.8	60%	Present
	Ulnar	5.5	57	61	58	22.3	50%	Present
	Tibial	20.4	41			65.6	50%	
	Peroneal	8.6	44			51.6	8.3%	
	Sural			14	44			
Case 4	Median	8.3	53	93	58	31.2	100%	
	Ulnar	9.4	54	66	64	32.0	100%	
	Tibial	25.7	41			57.0	100%	Present
	Peroneal	4.5	42			55.2	100%	Present
	Sural			16	41			
Case 5	Median	13.0	58	74	56	26.9	60%	
	Ulnar	7.5	54	39	52	28.6	100%	
	Tibial	12.1	39			62.2	62.5%	Present
	Peroneal	5.2	39			60.0	25%	Present
	Sural			15*	35*			
Case 6	Median	10.5	56	63	56	27.7	50%	Present
	Ulnar	9.8	56	44	52	26.0	80%	Present
	Tibial	12.3	43			52.0	100%	
	Peroneal	5.3	43			54.1	80%	
	Sural			16*	42*			

*The side with the more severe changes is listed; CMAP: compound motor action potential; SAP, sensory action potential, NCV, nerve conduction velocity

Notably, four patients experienced painful paresthesia, and three patients reported it as the initial presentation. The symptoms were often induced by heat or by exercise and subsided after resting or removal of the inducing factors. None of the patients reported significant limb deformities. There was no observable atrophy, fasciculation, or significant sensory loss.

### Electrophysiological studies of the PNS

The motor and sensory nerve conduction velocities were in the normal range in patients with sialidosis. Case 3 had a reduced CMAP amplitude in the right ulnar nerve, while case 5 had prolonged distal motor latency in the bilateral tibial and peroneal nerves. There were no repetitive CMAP observed. Five patients (83.3%) had abnormalities in the late response study, all of whom had prolonged F-wave latency, especially in the lower limbs (median nerve: 27.39 ± 1.27, 25.5–30.0 ms; ulnar nerve: 26.50 ± 1.91, 22.3–28.9 ms; peroneal nerve: 55.18 ± 9.00, 46.4–74.1 ms; tibial nerve: 54.76 ± 5.48, 46.2–62.2 ms). Repeater F-waves were noted in 3 patients. Three patients accepted the EMG study, and two patients had neurogenic polyphasic waves without spontaneous activities in sampled muscles. Sympathetic sensory response studies, done in 2 patients, revealed normal results. Only Case 3 received quantitative sensory testing, and the result is normal.

### A unique very late response other than F-wave was noted

**Fig. 1:**
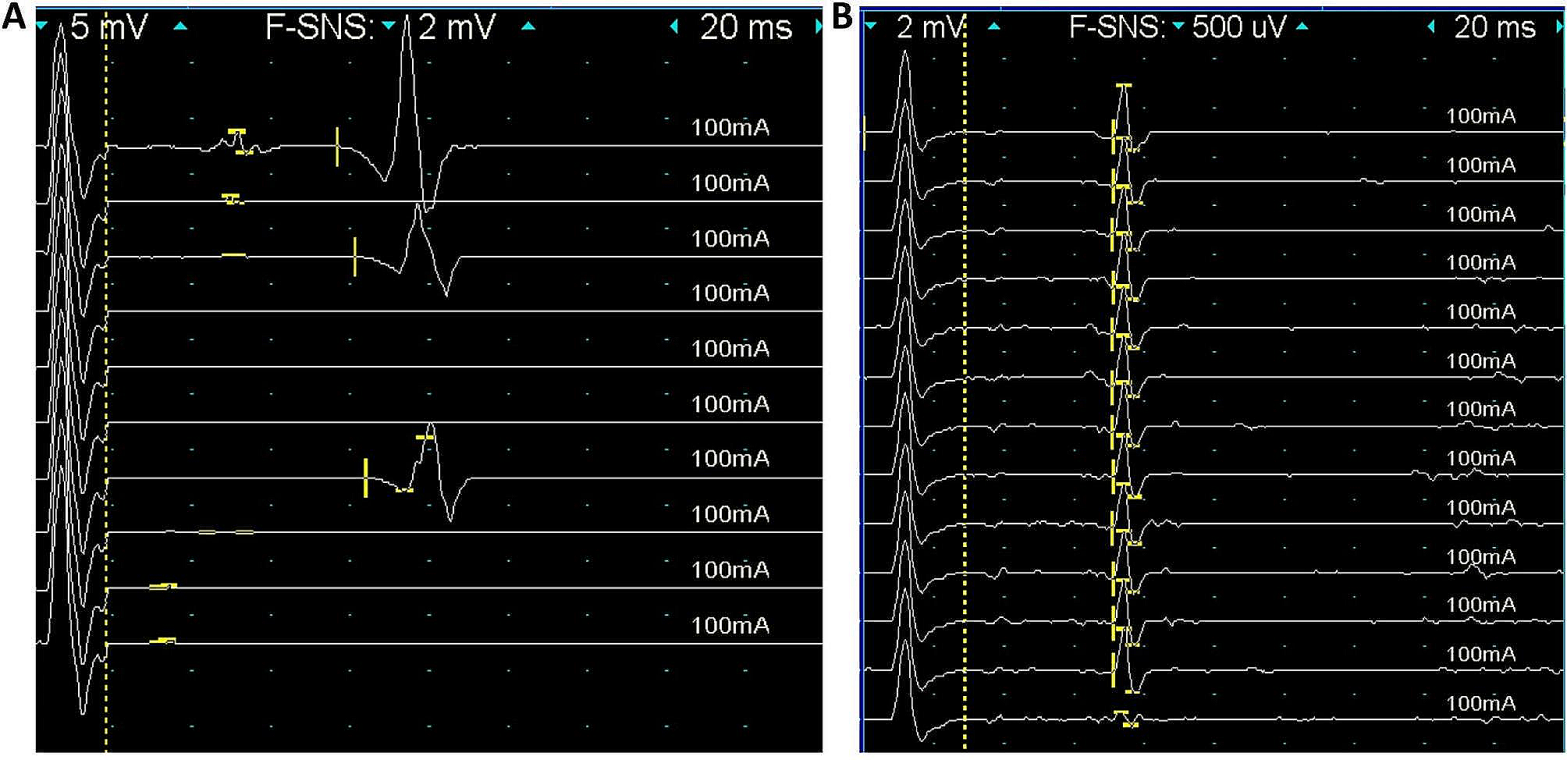
Findings from electrophysiological studies in patients with sialidosis. (**A**) In the F-wave study, very late responses (arrow) with a latency of 74 ms could be observed compared to the normal F waves with a latency of 42 ms (arrowhead). (**B**) These very late responses (latency: 70.8 ms) may exist without the presence of the F-wave

Beyond the typical F-waves, we also identified a special very late response in all of the recruited patients in the F-wave study. These responses have a latency that is much longer than the upper limit of F-wave latency in our lab (50 ms). The amplitude of these late responses ranged from 5 mV to 40 mV, and could present as either monophasic or multiphasic. The latency of the very late responses ranged from 70.8 to 176.4 ms. These very late responses could coexist with the F-wave (such as in patient 3, Fig. 1A) or appear when the F-wave was absent (Fig. 1B). This prolonged late response is most commonly presented in the tibial nerves, but could also be identified in the median and ulnar nerves.

## Discussion

Sialidosis is considered to be a disease with primary involvement of the CNS, and the involvement of PNS was rare reported. Our study revealed two intriguing findings: (1) the peculiar phenomenon in the late response study and (2) the painful paresthesia at the early stage of disease.

An intriguing finding in our study is the abnormalities in late response study, including the changes in the persistence and latency of F-wave, the presence of a repeater F-wave, and a “very late response” with high amplitude. The change in F-wave latency with normal motor conduction velocity is considered to correspond to a lesion in the proximal peripheral nerve or spinal cord. The repeater F-waves have been proposed to be related to lower motor neuron dysfunction or disinhibition due to upper motor neuron dysfunction [9–11]. The involvement of upper and lower motor neurons in previous autopsy studies supported that the change in F-waves was related to sialidosis [11, 12].

The presence of very late responses and giant waves has not been reported in previous studies. The difference in the shape and amplitude with normal F-waves, in addition to having a significantly prolonged latency, indicated that its pathology may be differ from traditional changes in F-waves [13, 14]. The proximal conduction time of the F wave could be calculated as 0.5*(F-M-1), with F being the latency of the F-wave, M being the distal latency, and the central conduction time estimated to be 1 millisecond. A very prolonged F-wave could be observed in Charcot-Marie-Tooth disease type 1, because the motor nerve conduction velocity is very slow. In the very late response observed in our patients, the latency was significantly prolonged compared with the estimated F-wave latency, which was calculated from the distal latency (M), motor nerve conduction velocity (MCV), and estimated distance between the stimulation and cord (D): $Estimated F \left(ms\right) =M+\frac{2D}{MCV}+1.$ Since the nerve conduction velocity and distal latency remained largely within normal limits, a prolonged central conduction time (much longer than 1 ms) is likely to be the cause of the changes. On the other hand, the origin of enlarged F-wave amplitude, previous studies had reported a possible relationship with lower motor dysfunction [15] although the involvement of the upper neurons could not be ruled out definitely [16]. Study on the myoclonus generator of sialidosis also revealed evidence indicating involvement of subcortical circuits, which result in motor hyperexcitability [17]. Considering that interneuron and upper motor neuron modify the excitability of lower motor neurons, we assume that the prolonged latency may result from aberrant interneuron cycles within the spinal cord, while the increased amplitude may be caused by disinhibition from the cortical-subcortical pathways, as had been reported by previous studies of sialidosis.

Our study revealed that patients may present with intermittent painful paresthesia as an initial symptom of sialidosis, which has rarely been described in previous studies. A case series documented that one of the 5 patients reported having painful neuropathy [18], while sensory deficits were found among 8 of 17 patients in another study [19]. In our study, all patients reporting these symptoms did not have continuous numbness or other negative symptoms. In the evaluation, all patients reported normal results on clinical sensory examination and quantitative sensory testing. As a result, this symptom should belong to positive symptoms, which may be related to ectopic activities or central disinhibition. In a previous pathological study, storage materials were noted in the Schwann cells of sural nerves [8]. Sialic acid accumulations had also been reported to be present in the dorsal root ganglion [12]. Combining the electrophysiological study of the peripheral nerve system that we provided, the origin of painful paresthesia may be the peripheral nerve. However, the hyperexcitability described above may also contribute to patients’ presence of painful paresthesia, for which the development of hyperalgesia and allodynia may be due to excessive responses and inadequate suppressive mechanisms to aberrant ectopic activities or benign sensory stimulations experienced by the patient [17].

Our study has several limitations. First, despite active recruitment of study subjects, we could only enroll 6 cases in our study. This is largely due to the rare nature of sialidosis, and study of a larger cohort with longer follow-up may reveal more abnormalities in the PNS for this disease. Second, many other techniques had been developed to further evaluate the pathophysiology of peripheral nerve disorders, such as nerve/skin biopsy, peripheral nerve imaging, and nerve excitability study. Incorporation of these techniques in future studies may lead to better understanding of the pathophysiology of this unique phenomenon in sialidosis.

## Conclusion

This study observed a novel very late response in the nerve conduction study of patients with sialidosis. This finding suggested that patients with sialidosis may present with involvement of the PNS, which is parallel with CNS involvement. The very late response may indicate abnormalities in the inhibition pathway in patients with sialidosis, warranting further studies.

## Methods

### Patients and clinical profiles

This retrospective study recruited patients with genetically confirmed sialidosis at National Taiwan University Hospital. All patients had detailed evaluations, including history taking, neurological examinations, image study, and electrophysiology studies, including electroencephalogram, somatosensory evoked potentials, visual evoked potentials, nerve conduction studies, F-wave studies, and electromyogram.

## Nerve conduction studies, F-wave studies, and electromyogram

Nerve conduction studies and electromyograms were performed using a Viking IV Electromyographer (Nicolet, Madison, WI) in all patients following established methods. The bilateral median, ulnar, tibial, peroneal, and sural nerves were studied. The results of nerve conduction studies were compared to normative data in our laboratory.

For the evaluation of the F-wave, the bilateral median, ulnar, tibial, and peroneal nerves were studied using the same equipment. Ten to 15 stimulations were applied at a frequency of 1 Hz. The minimal latency and persistence of F-waves were recorded. The minimal latency of the F wave was defined as the shortest latency of the presented F-wave as identified during the study. The persistence of the F-wave was defined as the percentage of definable F-waves in all stimulations. Repeater F waves were defined as F-waves with the same shape, latency, and amplitude as identified by the examiner. Electromyography was performed by a board-certified neurologist who determined the site for evaluation and interpretation of the results.

### Statistical analysis

Numerical variables are expressed as the mean ± SD. The minimal and maximal values were also included. All analyses were performed using Stata software (StataCorp LP, College Station, TX).

### Electronic supplementary material

Below is the link to the electronic supplementary material.

Supplementary Material 1

## Data Availability

The data that support the findings of this study are available from the corresponding author, upon reasonable request. The data are not publicly available due to the fact that the information contained could compromise the privacy of research participants. The data that support the findings of this study are available.
