# Supplementary material for: Unique clinical and electrophysiological features in the peripheral nerve system in patients with sialidosis – a case series study
Source: Orphanet J Rare Dis. 2024 May 24;19:217. doi: 10.1186/s13023-024-03216-8 (PMC11127318; doi:10.1186/s13023-024-03216-8)
Supplement: Supplementary file 1 — Supplementary Material 1 [file 13023_2024_3216_MOESM1_ESM.doc]

November 17, 2023

Professor Kazuo Abe

Editor

*Orphanet Journal of Rare Diseases*

Dear Professor Abe,

We are pleased to submit the revised manuscript entitled, *Unique Clinical and Electrophysiological Features in the Peripheral Nerve System in Patients with Sialidosis – a Case Series Study*, as a Research Article in *Orphanet Journal of Rare Diseases*. We have substantially revised the manuscript following the suggestions of the reviewers.

In addition to point-by-point responses, we have addressed the major concerns of the reviewers, including (1) the confirmation of diagnosis by genetic testing, and (2) selection of representative images. We have also revised the content of the Tables according to the suggestions of reviewers.

All changes in the revised manuscript are underlined and marked in red-colored font. The point-by-point revisions in response to each reviewer are stated in the following pages.

We thank the reviewers for their comments, which are extremely helpful and significantly improved the quality of this revised manuscript. We appreciate your considering the revised version of the manuscript.

Sincerely,

Hsueh Wen Hsueh, M.D., M.S.

Department of Neurology

National Taiwan University Hospital, Taipei, Taiwan

No.7, Chung Shan S. Road Taipei 100225 Taiwan

Phone: +886-2-23123456#262143

E-mail: b93401044@ntu.edu.tw

*Reviewer Comments:*

*Reviewer 1:*

*Reviewer #1: This manuscript reported an electrophysiological study of the patients with sialidosis.*

*The authors showed some abnormal findings, including prolonged F waves.*

*The introduction, results, and methods sections of the manuscript are clearly written.*

General response: Thank you for the positive comments.

*The authors need to solve the problems described below.*

*Subjects are patients with genetically-confirmed sialidosis.*

*However, in Table 1, the gene/enzyme activity for Case 3 is marked as "Pending."*

*Furthermore, the authors cited the electrophysiological findings of this case as the representative findings of this study. (shape).*

*Authors should explain the basis for this patient's diagnosis and why this patient was included as a subject.*

Response: Thank you for the comment. We have rechecked the data of the patient, and the study results showed decreased enzyme activities and pathogenic heterozygous mutation of NEU1. The original description is incorrect. This patient (Case 3) is indeed a Sialidosis patient with compatible genetic and enzymatic study findings. We have updated the findings in the table, as stated below.

**(Table 1, Genetic/Enzyme activity of patient 3)**

Genetic: c.544A>G (p.Ser182Gly), c.667_679del(p.Leu223GlufsTer76)

Enzyme activity: 0.09 nmol/mg Prot/hr

*minor:*

*The figure is unclear. Please clarify the figure.*

Response: Thank you for the comment. We have updated the figures. The updated figures are now re-uploaded to the submission system.
